# Supplementary material for: BAF60a deficiency uncouples chromatin accessibility and cold sensitivity from white fat browning
Source: Nat Commun. 2020 May 13;11:2379. doi: 10.1038/s41467-020-16148-1 (PMC7221096; doi:10.1038/s41467-020-16148-1)
Supplement: Supplementary file 1 — Supplementary Information [file 41467_2020_16148_MOESM1_ESM.pdf]

Supplementary Information

BAF60a deficiency uncouples chromatin accessibility and cold sensitivity from white fat browning

Liu et al.

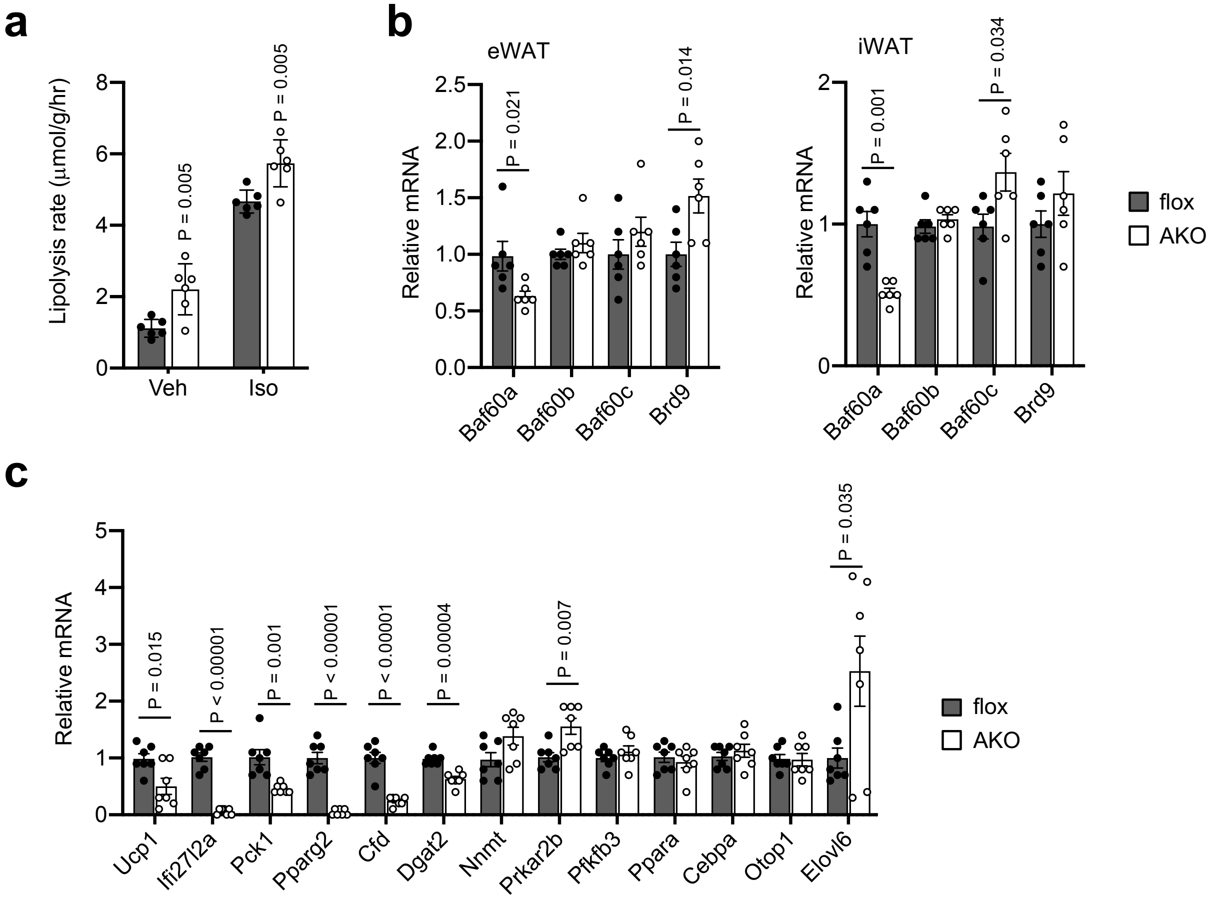

Supplementary Fig. 1. **a** Rate of lipolysis in ex vivo eWAT culture under basal (Veh) and stimulated (ISO) conditions (flox, n = 6; AKO, n = 6). Data represent mean ± sd. flox vs. AKO; two-tailed unpaired Student's t test. **b** qPCR analysis of gene expression in flox (n = 6, filled) and AKO (n = 6, open) mice kept at ambient room temperature. **c** qPCR analysis of BAT gene expression in flox (n = 7, filled) and AKO (n = 7, open) mice kept at ambient room temperature. Data in **b, c** represent mean ± sem; flox vs. AKO, two-tailed unpaired Student's t test. Source data are provided as a Source Data file.

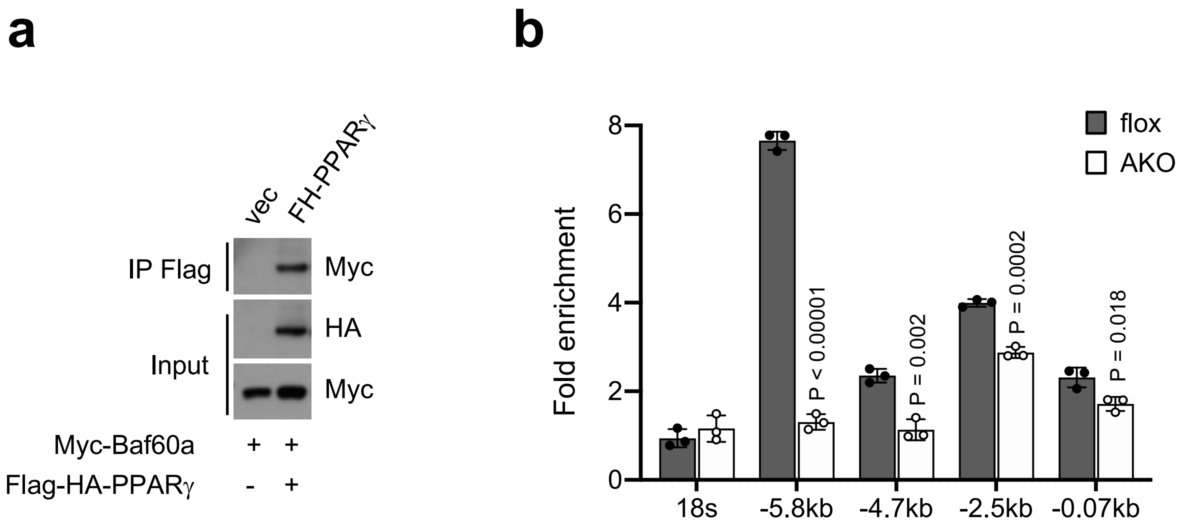

Supplementary Fig. 2: Impaired PPARγ binding to Ucp1 locus in BAF60a deficient BAT. a Coimmunoprecipitation of BAF60a and PPARγ in transiently transfected HEK293 cells (representative of three experiments). **b** ChIP-qPCR analysis of PPARγ binding on Ucp1 promoter regions in BAT from flox (n = 3, filled) and AKO (n = 3, open) mice. The locations of ChIP-qPCR primers are indicated by arrowheads in Fig. 2b. Data represent mean ± sd; flox vs. AKO, two-tailed unpaired Student's t test. Source data are provided as a Source Data file.

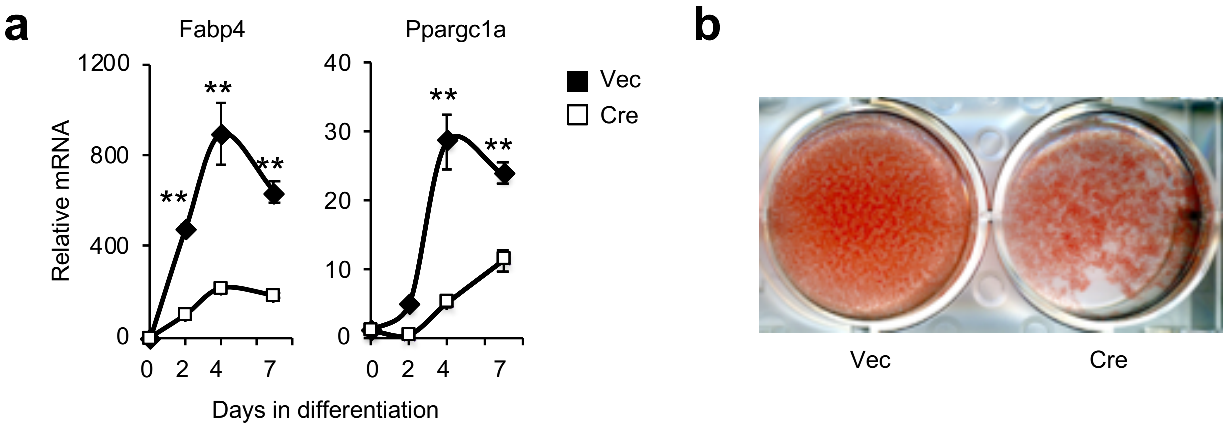

Supplementary Fig. 3: BAF60a is required for Brown adipocyte differentiation. BAT preadipocytes from BAF60a flox were transduced with vector (Vec) or a retroviral vector expressing Cre recombinase (Cre). **a** qPCR analyses of gene expression during adipogenesis (n = 3 per group). Data represent mean ± sd. **p < 0.01, Vec vs. Cre; two-tailed unpaired Student's t-test. **b** Oil Red-O staining of differentiated brown adipocytes (n = 3 per group). Source data are provided as a Source Data file.

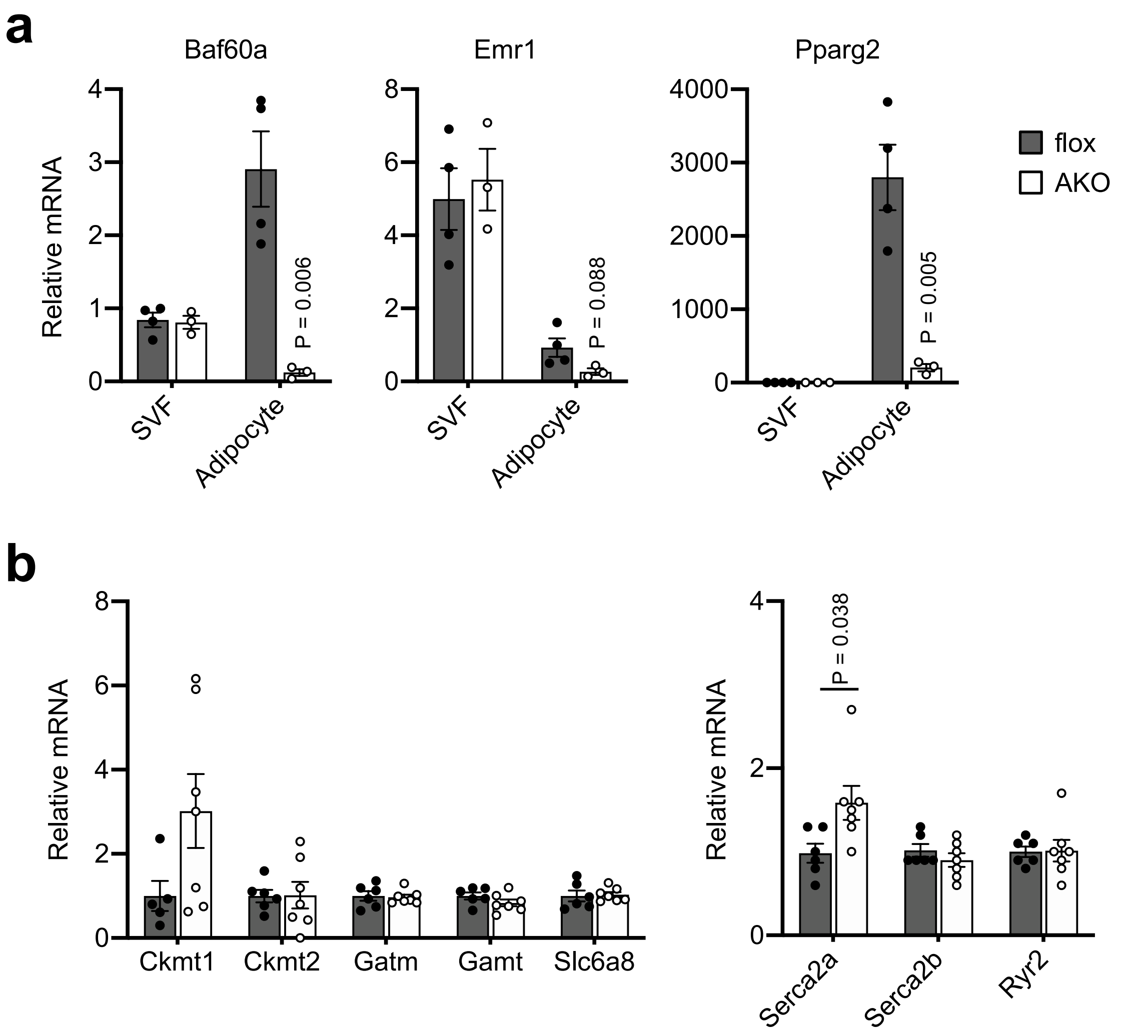

Supplementary Fig. 4. a qPCR analyses of gene expression in stromal vascular fraction (SVF) and mature adipocytes (Adipocyte) from iWAT of flox (n = 4, filled) and AKO (n = 3, open) mice. Note BAF60a was efficiently deleted in mature adipocytes but not SVF cells. **b** qPCR gene expression analyses for creatine metabolism (left) and calcium cycling (right) from iWAT of flox (n = 6, filled) and AKO (n = 7, open) mice after cold acclimation. Data represent mean ± sem. flox vs. AKO; two-tailed unpaired Student's t test. Source data are provided as a Source Data file.

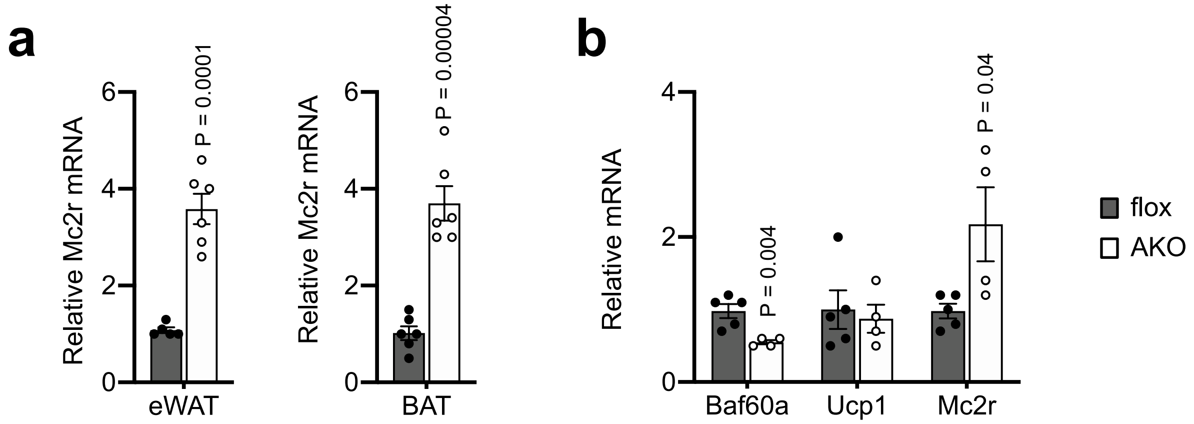

Supplementary Fig. 5: Expression of Mc2r is upregulated in BAF60a deficient adipose tissues. a qPCR analyses of Mc2r gene expression in eWAT (left) and BAT (right) of flox (n = 6, filled) and AKO (n = 6, open) mice. **b** iWAT qPCR gene expression in flox (n = 5, filled) and AKO (n = 4, open) mice after housing at 29°C for 13 days. Data represent mean ± sem. flox vs. AKO; two-tailed unpaired Student's t test. Source data are provided as a Source Data file.

Supplementary Table 1: list of qPCR primers and shRNAi oligos

Gene name	Forward primer	Reverse Primer
36B4	GAAACTGCTGCCTCACATCCG	GCTGGCACAGTGACCTCACACG
beta actin	CTCAGGGCATCGGAACCGCT	GGGAAATCGTGCGTGACATC
Baf60a	GGCGGTCCAAAATCGAAATC	ACCAGTTCCCGAATCCTTTG
Baf60b	GAAGCTGGACCAGACCATCG	CGCAGTTCCCGCATTATCTC
Baf60c	ACTATCAGCCTCCCCAGTTCAA	TGGGAGTCCTGTAGCCTGTTG
Brd9	TTGGAGATGGAAGTCTGCTCT	GCAACTTGCTAGACAGTGAACT
Mc2r	AGAACCAACATGAAGGGTGC	GGGTTATTTGGGCAGAAGGT
UCP1	GGCATTCAGAGGCAAATCAGCT	CAATGAACACTGCCACACCTC
Cidea	GCAGCCTGCAGGAACTTATCAGC	GATCATGAAATGCGTGTTGTCC
aP2	TGCCTTTGTGGGAACCTG	GCTTGTCACCATCTCGTTTTC
Elovl3	TGGACCTGATGCAACCCTAT	CCAACAACGATGAGCAACAG
Dio2	GATGCTCCCAATTCCAGTGT	TGAACCAAAGTTGACCACCA
Zbtb7b	CTCACCCATCCCTTGACCTA	CCAGCTCCTCTGGTGATAGC
Blnc1	CAAGGAAGTCATGAGCCCAATG	TAAAGGCTTCAACGGTGGCTG
PGC-1α	AGCCGTGACCACTGACAACGAG	GCTGCATGGTTCTGAGTGCTAAG
Ebf2	GGAACCGGAACGAGACCCCT	TCCCTTGGGTTTCCCGCTGT
Prdm16	CGGAAGAGCGTGAGTACAAATG	TCCGTGAACACCTTGACACAGT
Pparg1	GTGAGACCAACAGCCTGAC	TATCAGTGGTTCACCGCTTC
Pparg2	ATGGGTGAAACTCTGGGAGA	ATGGCATCTCTGTGTCAACC
Ppara	GCAGTGCCCTGAACATCGA	CGCCGAAAGAAGCCCTTAC
Oxtr	CACGGGTCAGTAGTGTCAAG	CACGATGAAGGCCAGAACAA
Avpr1a	CCTTTGTGATTGTAAGCGCC	GATCGTGGTGGAAGGGTTTT
Ghr	GGTCTTCTTAACCTTGGCACT	CTTTGCCAAGAGTAGCTGGT
Tshr	GGCTGGAACCACATTCTTTC	TGTCAAGGCATCAGGGTCTA
Prlr	ACGAAATGGGAAGCAGTACC	AAAGTCAGGTTCCGAGGAGG
Kiss1r	TGGTCGGAAACTCATTGGTC	GCAGCACAGTAGGAAAGTGA
Adrb3	GCTCAACAGGTTTGATGGC	AAGCTTCCTTGCTGGATCTTC
Chrna2	GCCACCGGAACCTATAACAGC	CCGGCGGATAACGAAGTAGTA
Cpt1b	ACAGACTTGCTACAGCACCTC	CGTCGAGGATTCTCTGGAAC
Acaa1b	ATGCTTCCATGCTGAGATTGT	TCCATCCTTGAAGGCAGGCTT
Acaa2	GATCTCAAGCTGGAAGATAC	ACCTCTGCTGAGACTGCAAG
Mcad	GCTGGAGACATTGCCAATCA	GGCGTCCCTCATCAGCTTCT
Acadl	TGCACACATACAGACGGTGCAG	TCAGATGCCCAGTATTTTGCC
Cox7a1	GTCTCCCAGGCTCTGGTCCG	CTGTACAGGACGTTGTCCATTC
Cox8b	TGGTTCCCAAAGCCCATGT	ATCCTGCTGGAACCATGAAGC
HK2	CCGCCGTGGTGGACAAGATA	AGCAGTGATGAGAGCCGCTC

Eno1	TGCGTCCACTGGCATCTAC	CAGAGCAGGCGCAATAGTTTTA
Pfkp	CGCCTATCCGAAGTACCTGGA	CCCCGTGTAGATTCCCATGC
Pkm2	ATCATTTGTACCATTGGGCCTG	TTCATTCCAGACTTAATCATCTCCTTC
Ldha	GGTGCATCCCATTTCCAC	GTCTGCGCTCTTCTTCAGG
Ldhb	GATTCACCCCGTGTCTACC	ATGGTCACATTGGAGGTTTG
Gapdh	TGGATTTGGACGCATTGGTC	TTTGCACTGGTACGTGTTGAT

shRNAMc2r	GCTCAGAGATGCATTCAAA	TTTGAATGCATCTCTGAGC

Supplementary Table 2: list of antibodies

Antibody name	Company	Catalog No	Species	Dilution
Ucp1	Alpha Diagnostic	UCP11-A	Rabbit	1:500 (for detection in differentiated cells)
Ucp1	Alpha Diagnostic	UCP11-A	Rabbit	1:5000 (for brown adipose tissue)
HSP90	Santacruz Biotech.	sc-13119	Mouse	1:1000
FASN	cell signaling	#3180	Rabbit	1:1000
FABP4	cell signaling	#3544	Rabbit	1:10,000
BAF60a	BD	611728	Mouse	1:1000
BAF60a	Proteintech	10998-2-AP	Rabbit	1:500
ACTIN	Sigma	A 4700	Mouse	1:2000
BRG1	Santacruz Biotech.	sc-10768	Rabbit	1:500
BRD9	Active Motif	61938	Rabbit	1:1000
BAF47	Bethyl	A301-087A	Rabbit	1:2500
PPARg	Santacruz Biotech.	sc-7273	mouse	1:1000
TH	abcam	ab112	rabbit	1:2000
pPKA substrate (RRXS/T)	cell signaling	#9624	Rabbit	1:2500
Myc	sigma	C3956	Rabbit	1:500
